# Clinical Significance of Tissue Factor Pathway Inhibitor 2, a Serum Biomarker Candidate for Ovarian Clear Cell Carcinoma

**DOI:** 10.1371/journal.pone.0165609

**Published:** 2016-10-31

**Authors:** Noriaki Arakawa, Hiroshi Kobayashi, Naohiro Yonemoto, Yusuke Masuishi, Yoko Ino, Hiroshi Shigetomi, Naoto Furukawa, Norihisa Ohtake, Yohei Miyagi, Fumiki Hirahara, Hisashi Hirano, Etsuko Miyagi

**Affiliations:** 1 Department of Medical Life Science, Graduate School of Medical Life Science, Yokohama City University, Yokohama, Kanagawa, Japan; 2 Advanced Medical Research Center, Yokohama City University, Yokohama, Kanagawa, Japan; 3 Department of Obstetrics and Gynecology, Nara Medical University, Nara, Japan; 4 Department of Biostatistics, Center for Novel and Explanatory Clinical Trials (Y-NEXT), Yokohama City University, Yokohama, Kanagawa, Japan; 5 Bioscience Division, Reagent Development Department, Tosoh Corporation, Ayase, Kanagawa, Japan; 6 Research Institute, Kanagawa Cancer Center, Yokohama, Kanagawa, Japan; 7 Yokohama City University Hospital, Department of Obstetrics and Gynecology, Yokohama, Kanagawa, Japan; University of Nebraska Medical Center, UNITED STATES

## Abstract

**Background:**

There is currently no reliable serum biomarker for ovarian clear cell carcinoma (CCC), a highly lethal histological subtype of epithelial ovarian cancer (EOC). Previously, using a proteome-based approach, we identified tissue factor pathway inhibitor 2 (TFPI2) as a candidate serum biomarker for CCC. In this study, we sought to evaluate the clinical diagnostic performance of TFPI2 in preoperative prediction of CCC.

**Methods:**

Serum TFPI2 levels were measured in serum samples from a retrospective training set consisting of patients with benign and borderline ovarian tumors, EOC subtypes, and uterine diseases. Via receiver operating characteristic (ROC) analyses, we compared the diagnostic performance of TFPI2 with that of CA125 in discrimination of patients with ovarian CCC from other patient groups. The observed diagnostic performances were examined in a prospective validation set.

**Results:**

The 268-patient training set included 29 patients with ovarian CCC. Unlike CA125, which was also elevated in patients with endometriosis and several EOC subtypes, serum TFPI2 levels were specifically elevated only in ovarian CCC patients, consistent with the mRNA expression pattern in tumor tissues. The area under the ROC curve (AUC) of serum TFPI2 was obviously higher than that of CA125 for discrimination of CCC from other ovarian diseases (AUC = 0.891 versus 0.595). Applying a cut-off value of 280 pg/mL, TFPI2 could distinguish early-stage (FIGO I and II) CCC from endometriosis with 72.2% sensitivity, 93.3% specificity, and 88.8% accuracy. Similar results were confirmed in an independent 156-patient prospective validation set.

**Conclusions:**

TFPI2 is a useful serum biomarker for preoperative clinical diagnosis of CCC.

## Introduction

Epithelial ovarian cancer (EOC) is one of the major causes of death from gynecological malignancies [1]. EOC is a heterogeneous disease with multiple histological subtypes, including serous, mucinous, endometrioid, and clear cell carcinoma (CCC). CCC is a rare subtype with an incidence of <5% of all EOC in Western countries. By contrast, in Japan, the incidence of CCC has been increasing, representing >20% of cases [2]. The clinical and biological characteristics of CCC differ from those of other EOC subtypes. For example, CCC is often chemoresistant, leading to poorer prognosis; in particular, its response rate to paclitaxel plus carboplatin therapy (22–56%) is lower than those of other histological types [3]. The survival rates of patients with advanced CCC are significantly lower than those of patients with advanced serous-type EOC [4]. Therefore, earlier diagnosis of CCC and monitoring of CCC are vital.

Cancer antigen 125 (CA125, also known as carbohydrate antigen 125), the most widely used serum biomarker for EOC, was discovered over 30 years ago [5]. The major disadvantages of CA125 include poor sensitivity and specificity for EOC, especially for diagnosis of early-stage disease [6]. CA125 is elevated in the serum of >80% of patients with high-grade serous adenocarcinoma, but false-negative results for CA125 frequently occur in cases of stage I/II EOC [7] and CCC [8]. However, CA125 is the only currently used diagnostic biomarker for ovarian CCC. Furthermore, CA125 is also elevated in patients with endometriosis (EMS), from which CCC is thought to arise [9, 10]. Although it is very important to monitor the transformation from EMS into CCC, there is currently no biomarker that can efficiently distinguish between these diseases. Therefore, timely diagnosis of CCC requires development of more accurate biomarkers.

In a previous study using a proteomics approach, we showed that a serine protease inhibitor, tissue factor pathway inhibitor 2 (TFPI2, also known as placental protein 5), was specifically present in conditioned media from CCC-derived cell lines [11]. Using a highly sensitive and specific automated ELISA system that we developed, we found that serum levels of TFPI2 were higher in patients with CCC relative to those in normal healthy controls and patients with EMS [11]. In this study, we evaluate in detail the performance of TFPI2 as a serum biomarker for preoperative diagnosis for CCC, using a retrospective training data set and a prospective validation data set.

## Materials and Methods

### Patient population and sample collection

In this study, we assembled two independent patient groups, the retrospective training set and the prospective validation set, in which we separately evaluated the diagnostic performance of TFPI2. Both sets consisted of patients with various ovarian neoplasms or uterine neoplasms prior to surgery. Patients who were treated with neoadjuvant chemotherapy and pregnant women were excluded. This study was performed in accordance with the Declaration of Helsinki, and approved by the Yokohama City University Hospital Institutional Review Board and the Nara Medical University Ethics Committee. All patients provided written informed consent. We also followed the Standards for Reporting of Diagnostic Accuracy (STARD) statement guideline [12].

The training set was retrospectively assembled from patients histologically diagnosed with gynecological disease at two clinical institutions in Japan: 143 patients in Yokohama City University (March 2006 –April 2012) and 125 patients in Nara Medical University (June 2008 –May 2013). Overall, 268 samples were included, of which 114 were diagnosed as benign ovarian tumor, 8 were from borderline ovarian tumor (low malignant potential), 108 were from EOCs, and 38 were from uterine diseases.

The independent validation set was prospectively assembled from 156 patients who were diagnosed with an adnexal mass by ultrasound, CT scan, or MRI, and then prior to suspected ovarian neoplasm in Yokohama City University between April 2012 and April 2014. The validation set was assembled and processed before disease status was known. Therefore, the distributions of benign ovarian diseases, borderline ovarian diseases, and each EOC subtype in the validation set were thought to reflect the distributions seen in clinical practice.

Serum samples were collected at the pre-surgery stage after the pelvic mass was confirmed, and surgery was scheduled within 10 days of blood collection. The blood was drawn into the Venoject II serum separator tube (VP-AS109K60, Terumo, Tokyo, Japan), centrifuged, aliquoted, and stored at -80°C until analysis was carried out. After surgery, disease status was subsequently examined using excised tissue by experienced pathologists for diagnosis, histological analysis and staging (I-IV), according to WHO 2003 and International Federation of Gynecology and Obstetrics (FIGO 1988) standards, respectively, before both classifications were revised in 2014.

### Serum TFPI2 assay

Using two anti-TFPI2 monoclonal antibodies, the TFPI2 concentration in serum samples was measured by a sandwich-type, one-step immunofluorometric assay on an automated immunoassay analyzer (AIA) system (TOSOH, Japan), as described previously [11]. The AIA system performs automated sample dispensation, incubation of the reaction cup, bound/free washing procedure, dispensation of 4-methylumbelliferyl phosphate substrate, fluorescence detection, and reporting of results. All reagents in the AIA system were manufactured at TOSOH diagnostics product divisions. Briefly, antibody-coated magnetic beads (produced in-house) and alkaline phosphatase-labeled antibody were lyophilized with assay buffer containing 5% BSA, 5% sucrose, 10 mM MgCl_2_, 100 mM Tris–HCl (pH 7.5) in the reaction cup. As the calibration standard, recombinant TFPI2 protein (R&D Systems) was spiked into fetal bovine serum. Serum samples or the calibration standards were pre-diluted 2.5-fold in sample dilution buffer containing 140 mM NaCl and 10 mM Tris-HCl (pH 7.4). After setting samples and the reaction cup on the AIA machine, 50 μL of diluted samples or standards were automatically applied to each reaction cup, and then the fluorescence was measured. This method, called the Pre-Diluted Assay, was used to analyze serum samples from the training and validation sets.

In addition, we developed an improved method (Direct Assay) that can more simply quantify and rapidly detect serum TFPI2 levels without pre-dilution of serum samples. The Direct Assay is currently very close to a final product suitable for clinical use. This method uses modified compositions of calibration standard-diluent buffer and assay buffer, but the same monoclonal antibodies as the Pre-Diluted Assay. To determine whether TFPI2 also exhibits good diagnostic performance in the Direct Assay, a subset of the training and validation sets (training set-2 and validation set-2) were analyzed using this method.

The performance of each assay was confirmed to meet the following criteria: within-run precision coefficient of variation (CV) less than 5%; measurement within the linear standard range; R^2^ of the standard curve regression line at least 0.95; dilution linearity (CV) less than 5%; spike and recovery over 90%; and no cross-reactions against human tissue factor pathway inhibitor (TFPI), a known structural homologue of TFPI2.

### Statistical analysis

Serum TFPI2 levels measured by the AIA system and serum CA125 levels at the time of sample collection were presented as box-and-whisker plots. The nonparametric Mann-Whitney *U* test was used to evaluate differences between two groups. ROC curves were constructed for serum TFPI2 and CA125 by plotting sensitivity *versus* (100–specificity), and the areas under the ROC curves were calculated when discriminating patients with CCC (1) from patients with other ovarian diseases, (2) from patients with borderline and patients with other subtype EOCs, and (3) from patients with EMS. The cutoff values of TFPI2 were predefined using the Youden index based on the training data set. Sensitivity, specificity, predictive and negative predictive values, and accuracy were determined for the predefined cutoff values. Because TFPI2 is a placental protein whose levels increase in the serum of pregnant women, the correlation between serum TFPI2 levels and age or menstruation cycles were assessed using the Spearman correlation coefficient [13]. All data were analyzed using the GraphPad PRISM software (version 5.0) and Microsoft Excel software.

## Results

### Serum TFPI2 levels in gynecological diseases in the training set: comparison with CA125

To evaluate the diagnostic power of TFPI2, serum samples from a total of 268 patients (230 ovarian diseases and 38 uterine [Ut] diseases) were retrospectively collected from two institutions, as described in Tables 1 and 2, and used as the training set. Among the patients with ovarian disease, there were 114 cases of benign tumors (mean age [range]; 47.5 [22–81] years), 8 cases of borderline tumors (55.8 [31–75] years), 29 cases of CCC (56.7 [37–79] years), and 79 cases of non-CCC EOCs (55.0 [15–84] years).

**Table 1:** Sources of samples.

Histology	Serum Samples		
	Training Set		Validation Set
	YCU	NMU	YCU
Benign ovarian tumor	56	58	40
Non-EMS	43		23
EMS	13	58	17
Borderline ovarian tumor	7	1	15
Ovarian cancer	42	66	38
CCC	11	18	7
Serous	10	17	17
Endometrioid	7	21	4
Mucinous	5	3	4
Other	9	7	6
Uterine disease	38		58
Uterine fibroids	5		17
Cervical cancer	18		15
Endometrial cancer	15		26
Other disease			5
Total	143	125	156

Abbreviations: YCU, Yokohama City University; NMU, Nara Medical University; EMS, endometriosis.

**Table 2:** Clinicopathologic characteristics of the patients.

Disease	Histology	Training set (n = 268)									Validation set (n = 156)								
		No. of Patients	FIGO (No. of patients)					Age (years)			No. of Patients	FIGO (No. of patients)					Age (years)		
			I	II	III	IV	unknown	Range	Median	Mean		I	II	III	IV	unknown	Range	Median	Mean
Ovarian disease		230									93								
	Benign tumor	114						22–81	44.5	47.5	40						20–76	42.5	42.8
	EMS	71						28–71	43	44.2	17						23–57	44	41.4
	non-EMS	43						22–81	54	52.9	23						20–76	41	43.9
	Borderline tumor	8	8	0	0	0	0	31–75	53	55.8	15	14	0	1	0	0	16–80	46	47.6
	Ovarian cancer	108									38								
	CCC-subtype	29	15	3	8	3	0	37–79	57	56.7	7	3	1	3	0	0	38–71	58	57.7
	non-CCC-subtypes	79	22	12	25	16	4	15–84	57	55.0	31	8	4	11	6	2	27–84	63	61.5
	Serous	27	3	2	14	8	0	15–79	58	55.2	17	3	2	9	2	1	36–76	65	63.3
	Endometrioid	28	11	8	6	1	2	35–84	55	56.6	4	0	2	1	1	0	61–77	65	67.0
	Mucinous	8	5	1	0	2	0	24–77	47.5	47.1	4	3	0	0	1	0	27–75	46.5	48.8
	Other	16	3	1	5	5	2	25–75	60	56.8	6	2	0	1	2	1	37–84	62	61.2
Uterine disease		38						28–88	47	49.8	58						26–85	47	50.7
	Uterine fibroids	5						35–70	40	44.8	17						28–66	46	46.2
	Cervical cancer	18	7	7	0	1	3	28–65	40	42.8	15	11	3	0	0	1	26–70	46	47.6
	Endometrial cancer	15	8	2	1	4	0	35–88	60	51.4	26	15	2	6	1	2	38–85	53	55.7
Other disease		0									5						33–66	60	55.4
	Total	268									156								

Abbreviations: EMS, endometriosis.

**Fig 1:**
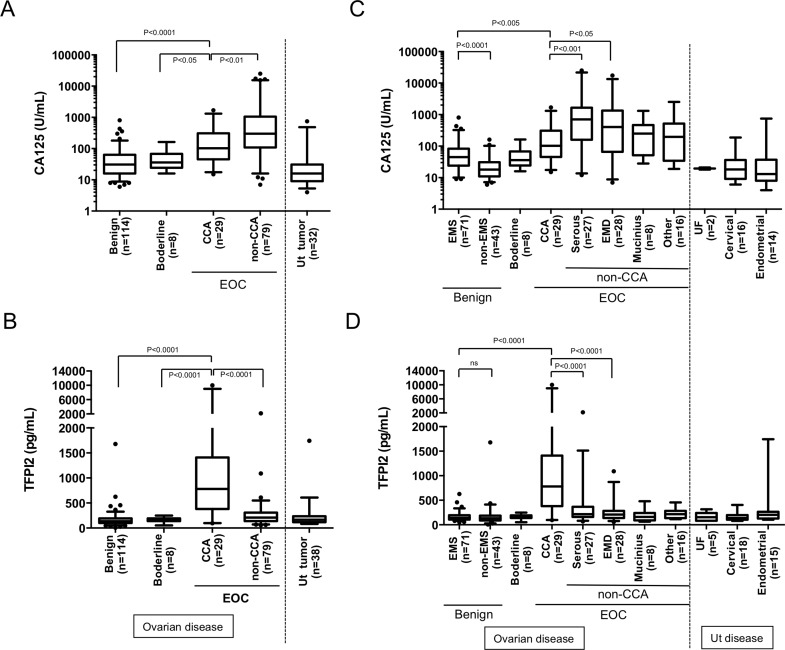
Distribution of CA125 and TFPI2 in serum samples from patients with CCC and other gynecological diseases (retrospective training set, n = 268), along with TFPI2 mRNA levels in their tissue samples. Serum CA125 levels in patients with CCC, other ovarian diseases, and uterine (Ut) diseases (**A**). Distribution of CA125 levels at the time of sample collection. Serum TFPI2 levels in patients with CCC, other ovarian diseases, or Ut diseases in the training set (**B**). Serum TFPI2 levels in samples of the training set were measured by the Pre-Diluted Assay as described in Materials and Methods. Comparison of CA125 (**C**) and TFPI2 (**D**) levels in patients with EMS, non-EMS, each EOC subtypes, and various Ut tumors (UF: uterine fibroids, cervical cancer, endometrial cancer). Box plots display 5^th^, 25^th^, 50^th^ (median, middle horizontal line), 75^th^, and 95^th^, percentiles. Statistical analysis was performed by nonparametric Mann-Whitney *U* test.

Expression levels of TFPI2 in the training set were measured on an AIA system and compared to serum CA125 levels, which were examined before surgery. CA125 levels tend to be higher in patients with CCC and other EOC subtypes than in patients with benign ovarian tumors, borderline ovarian tumors, or Ut disease; consequently, CA125 is the gold-standard biomarker for EOC (Fig 1A and Table 3). However, serum CA125 levels tended to be lower in CCC patients (median [interquartile range], 102.0 [45.5–309.5] U/mL) than in patients with non-CCC subtype EOCs (301 [109–1059] U/mL), as reported previously [14, 15] By contrast, serum TFPI2 levels were significantly (Mann–Whitney test; *P* < 0.0001) elevated in CCC patients (781.8 [381.4–1410] pg/mL) relative to patients with benign ovarian tumors (136.4 [99.93–191.0] pg/mL), borderline ovarian tumors (151.3 [137.7–192.1] pg/ml), or non-CCC subtype EOCs (208.5 [139.1–307.3] pg/ml). Serum TFPI2 levels were also significantly higher in CCC patients than in patients over 50 years old with benign ovarian tumors (data not shown).

**Table 3:** Serum levels of TFPI2 and CA125 in the training and validation sets.

Biomarker	Groups in training set (n)							
	EMS (n = 71)	non-EMS (n = 43)	BD (n = 8)	Total CCC (n = 29)	Early CCC (n = 18)	Late CCC (n = 11)	non-CCC EOCs (n = 79)	Ut Diseases (TFPI2, n = 38; CA125, n = 32)
TFPI2 (pg/mL)	137.7 (104.0–191.0)	132.3 (86.2–186.7)	151.3 (137.7–192.1)	781.8 (381.4–1410)	484.8 (226.3–1044)	1142 (716.0–1796)	208.5 (137.7–307.3)	162.2 (115.3–231.8)
CA125 (U/mL)	45.0 (24–83)	18.1 (11–31)	36.0 (24.3–67.75)	102 (45.5–309.5)	74.0 (28.8–151.75)	181.0 (102.0–465.0)	301 (109–1059)	16 (9–30.75)
Biomarker	Groups in validation set (n)							
	EMS (n = 17)	non-EMS (n = 23)	BD (n = 15)	Total CCC (n = 7)	Early CCC (n = 4)	Late CCC (n = 3)	non-CCC EOCs (n = 31)	Ut Diseases (TFPI2, n = 58; CA125, n = 42)
TFPI2 (pg/mL)	182.1 (151.1–213.0)	151.1 (88.9–182.1)	151.1 (120.1–213.0)	518.3 (182.1–934.7)	335.1 (135.6–690.4)	934.7 (518.3–2197)	213.0 (151.1–305.4)	182.1 (120.1–243.9)
CA125 (U/mL)	58 (36.5–90.5)	17 (11–25)	43 (25–80)	352 (100–1143)	621.5 (37.0–3472)	352.0 (123.0–518.0)	95 (51–419)	18.5 (11.25–36)

All tested values are expressed as the median (interquartile range). Abbreviations: EMS, endometriosis; BD, borderline ovarian tumor.

Fig 1C and 1D shows serum levels of TFPI2 and CA125 in patient populations stratified as follows: benign ovarian tumors were divided into EMS and non-EMS; EOCs were divided according to histological subtype; and uterine tumors were divided into uterine fibroids, cervical cancer, and endometrial cancer. Of patients with benign ovarian tumors, 71 cases were classified as endometriosis (EMS), and 43 cases as non-EMS benign ovarian tumors. Of patients with non-CCC EOCs, 27 cases had serous-type EOC, 28 cases had endometrioid–type EOC, 8 cases had mucinous-type EOC, and 16 cases had EOCs of other subtypes.

In patients with benign ovarian diseases, serum CA125 levels were higher in patients with EMS (45 [24–83] U/mL) than in patients with non-EMS tumors (18 [11–31] U/mL), consistent with previous reports [7, 16]. Unlike CA125, which was also elevated in other subtype EOC patients, serum TFPI2 levels was specifically elevated only in ovarian CCC patients, although they were slightly higher in patients with serous-type EOC (216.7 [159.5–368.0] pg/ml) and patients with endometrioid-type EOC (208.6 [137.7–283.8] pg/ml). Consistent with the serum TFPI2 levels, real-time RT-PCR analysis showed that the CCC-specific expression of TFPI2 mRNA also occurred in tissue specimens (S1 Fig). Low expression of TFPI2 mRNA was found in tumor tissues of CCC patients with lower serum TFPI2 levels. These findings indicate that TFPI2 gene expression in ovarian tumor tissue is responsible for elevation of serum TFPI2 in CCC patients.

### Diagnostic accuracy of TFPI2 for CCC prediction in the training set

**Fig 2:**
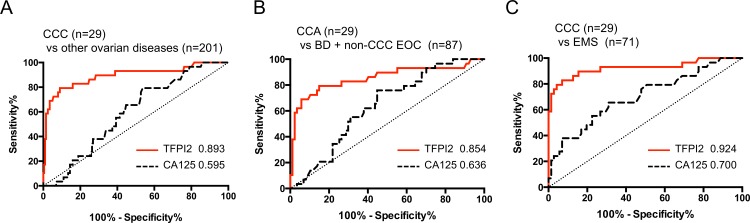
**ROC and AUC values for serum CA125 and TFPI2 levels in discrimination of CCC from other ovarian diseases (benign diseases, borderline, and non-CCC EOCs) (A), CCC versus borderline ovarian tumors (BD) and non-CCC EOCs (B), and CCC versus EMS (C).** Red line, TFPI2; black dotted line, CA125. Numbers indicate the calculated AUC values for TFPI2 and CA125.

To evaluate the diagnostic performance of serum TFPI2 for prediction of CCC among the different patient groups, we performed receiver operating characteristic (ROC) analyses, and then compared the area under the ROC curves (AUCs) for TFPI2 and CA125. The ROC analysis revealed that TFPI2 was obviously superior to CA125 in discrimination of CCC patients from patients with other ovarian diseases (AUC = 0.891 versus 0.595), borderline ovarian tumors and non-CCC EOCs (AUC = 0.856 versus 0.639), and EMS, respectively (AUC = 0.920 versus 0.700; Fig 2A–2C and Table 4). These AUC values of TFPI2 were also higher than those of CA125 in distinguishing all EOC patients (including CCC patients) from patients with benign ovarian tumors (AUC = 0.814; Table 4). These results indicated that TFPI2 might be suitable for diagnosis of CCC patients.

**Table 4:** Comparison of performances of TFPI2 and CA125 in discriminating CCC patients from other patient groups.

Discrimination	Biomarker	Sample set	No. of samples (No. of CCC)	AUC	95% CI	Cut-off value	Sensitivity (%)	Specificity (%)	PPV (%)	NPV (%)	Accuracy (%)
CCC vs other ovarian diseases	TFPI2	Training set	230 (29)	0.893	0.812 to 0.972	345 pg/mL	79.3	91.0	56.1	96.8	89.6
		Validation set	93 (7)	0.817	0.597 to 1.000		71.4	91.9	45.5	97.5	90.3
	CA125	Training set	230 (29)	0.595	0.501 to 0.688	35 U/mL	79.3	41.8	16.9	93.3	46.5
		Validation set	93 (7)	0.785	0.589 to 0.981		85.7	43.0	12.8	97.4	46.2
CCC vs BD + other EOCs	TFPI2	Training set	116 (29)	0.854	0.761 to 0.952	345 pg/mL	79.3	85.1	63.9	92.5	83.6
		Validation set	53 (7)	0.797	0.558 to 1.035		71.4	85.7	50.0	94.7	83.7
	CA125	Training set	116 (29)	0.639	0.533 to 0.745	35 U/mL	79.3	18.4	25.6	72.7	33.6
		Validation set	53 (7)	0.708	0.493 to 0.923		85.7	19.0	18.8	88.9	28.6
CCC vs EMS	TFPI2	Training set	100 (29)	0.924	0.843 to 0.997	280 pg/mL	82.8	93.0	82.8	93.0	90.0
		Validation set	24 (7)	0.807	0.561 to 1.052		71.4	100	100	89.5	91.7
	CA125	Training set	100 (29)	0.700	0.584 to 0.817	35 U/mL	79.3	43.7	36.5	83.8	54.0
		Validation set	24 (7)	0.803	0.545 to 1.060		85.7	23.5	31.6	80.0	41.7
All EOCs vs BN	CA125	Training set	230 (116*)	0.814	0.760 to 0.869	35 U/mL	81.0	55.3	64.8	74.1	68.3
		Validation set	93 (53*)	0.761	0.664 to 0.857		75.5	62.5	72.7	65.8	69.9

Abbreviations: BD, borderline ovarian tumor; EMS, endometriosis; BN, benign ovarian tumor. Asterisks indicate numbers of EOC patients.

Based on the ROC analysis in the training set, we predefined the cutoff values of TFPI2 for predicting patients with CCC. When we set the cutoff value for discriminating CCC patients from patients with other ovarian diseases at 345 pg/mL, the point with the highest Youden Index, diagnostic sensitivity and specificity were 79.3% and 91.0%, and positive predictive value (PPV), negative (NPV) predictive values, and accuracy were 56.1%, 96.8%, and 89.6%, respectively (Table 4). Among patients with non-CCC gynecological disease (n = 239), TFPI2 levels > 345 pg/mL were observed in five patients with benign ovarian tumors (3 EMS and 2 non-EMS), 13 patients with non-CCC subtype EOCs (7 serous, 3 endometrioid, 1 mucinous, and 2 other-subtypes), and 5 patients with uterine disease (2 cervical cancer, 3 endometrioid endometrial cancer) (S1 Table).

We also set 345 pg/mL as the cutoff value for discriminating CCC patients from patients with BD and non-CCC EOCs, and 280 pg/mL for discriminating from patients with EMS. In each case, the diagnostic performance of TFPI2 for predicting CCC was superior to that of CA125 at a cutoff value of 35 U/mL, which is typically used in general clinical fields (Table 4).

### Validation using independent sample set

Next, we investigated whether our results from the training set were reproducible by evaluating the diagnostic potential of serum TFPI2 for CCC in an independent group (validation set), collected prospectively for 2 years at Yokohama City University. The 156 patients (93 patients with suspected ovarian cancer and 58 with other uterine diseases) were enrolled in the validation set. On the basis of histological diagnosis after surgery, 38 cases were classified as EOCs; 18.4% (7/38) were diagnosed as CCC, 44.7% (17/38) as serous-type, and 10.5% (4/38) were with endometrioid- and mucinous-type EOCs for both (Table 2). The distribution of EOC subtypes was similar to the typical incidence rate of these subtypes among Japanese women [2].

Consistent with the results obtained from the training set, and in contrast to CA125, the TFPI2 level in patients with CCC (median [interquartile range], 518.3 [182.1–934.7] pg/ml) was elevated relative to that in patients with EMS (182.1 [151.1–213.0] pg/ml), non-EMS (151.1 [88.9–182.1] pg/ml), borderline ovarian tumors (151.1 [120.1–213.0] pg/ml), or non-CCC EOCs (213.0 [151.1–305.4] pg/ml; Table 3 and S2 Fig).

AUC values also indicated that TFPI2 was superior to CA125 in discriminating CCC patients from patients with other ovarian diseases (AUC = 0.817 versus 0.785), BD and non-CCC EOCs (AUC = 0.797 versus 0.708), or EMS (AUC = 0.807 versus 0.803; Table 3). Applying the cutoff values predefined in the training set, the diagnostic performance values of TFPI2 for discriminating CCC patients from other patient groups were similar to the results obtained from the training set (Table 3). Furthermore, as seen in the training set, TFPI2 levels >345 pg/mL were also observed in a subset of patients with serous-type EOC (5 of 17) or endometrioid-type endometrial cancer (2 of 26) (S1 Table).

### Validation using an improved assay method

In addition, we validated the clinical utility of TFPI2 using another assay system, the Direct Assay, described in Materials and Methods. Using the Direct Assay, we analyzed a subset of samples from the training set (123 patients with ovarian disease; training set-2), and the validation set (78 patients with ovarian disease; validation set-2). Regression analysis of TFPI2 values obtained with the Pre-diluted Assay and the Direct Assay revealed a strong positive correlation (correlation coefficient, r = 0.933); the slope of the regression line (*b*) was 0.825 ± 0.0195, significantly less than 1 (p < 0.0001, df = 129) (S3 Fig). Applying the cutoff values for the Direct Assay calculated using training set-2, 270 and 220 pg/mL, the diagnostic performance values for TFPI2 were very similar to those obtained using the Pre-Diluted Assay (S2 Table).

### Evaluation of TFPI2 in early- and late-stage CCC

**Fig 3:**
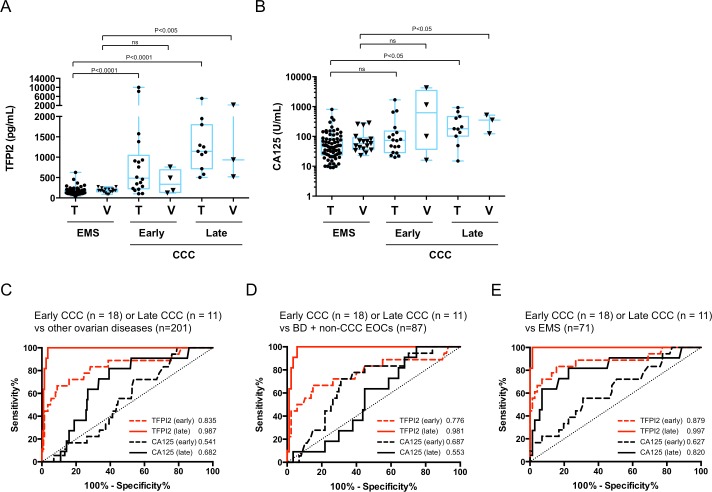
Distributions and AUC values for TFPI2 and CA125 in a comparison of CCC patients at early and late stages. CCC patient groups in training and validation sets were divided into early (FIGO I/II) and late (FIGO III/IV) stages. Distributions of serum levels of TFPI2 (**A**) and CA125 (**B**) in patients with EMS and patients with CCC at early and late stages in the training and validation sets (T, training set; V, validation set). ROC curves and AUC values for serum CA125 and TFPI2 levels in discrimination of patients with early- or late-stage CCC from other ovarian diseases (benign diseases, borderline, and non-CCC EOCs) (**C**), of borderline from non-CCC EOCs (**D**), or of CCC from EMS (**E**) in the training set. Red line and red dotted line indicate ROC curves for TFPI2 in discrimination of early- and late-stage CCC patients, respectively. Black line and black dotted line indicate ROC curves for CA125 in discrimination of early- and late-stage CCC, respectively. Numbers indicated calculated AUC values.

We evaluated the serum levels of TFPI2 and CA125 in CCC patients according to FIGO tumor stages. Of patients with CCC in the training and validation sets, 62% (18/29) and 57% (4/7) were at early stage (FIGO I/II), whereas 38% (11/29) and 43% (3/7) were at late stage (FIGO III/IV), respectively. These tumor stage distributions were in line with the incidence rate of CCC reported in the literature [17, 18]. Compared to CA125, obvious increases in serum TFPI2 level were observed during tumor progression in both the training and validation sets (Fig 3A and 3B and Table 3). Fig 3C–3F shows ROC curves for TFPI2 and CA125, comparing patients with early- and late-stage CCC from different disease groups in the training set. AUCs of TFPI2 were higher than those of CA125 in discrimination of patients with early-stage CCC from other ovarian diseases (AUC = 0.835 versus 0.541), borderline ovarian tumors and other EOCs (AUC = 0.776 versus 0.687), and EMS (AUC = 0.879 versus 0.627), suggesting that unlike CA125, TFPI2 could discriminate patients with early-stage CCC and other patient groups. Furthermore, TFPI2 could almost completely discriminate patients with late-stage CCC from those with other ovarian diseases (AUC = 0.987), borderline ovarian tumors and other EOCs (AUC = 0.981), or EMS (AUC = 0.997).

### Correlation between TFPI2 and CA125

**Fig 4:**
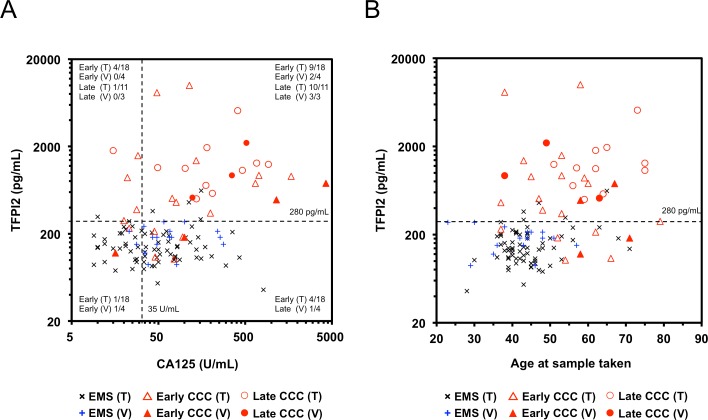
Correlation of serum TFPI2 levels vs. serum CA125 levels and age in CCC and in EMS patients. Two-dimensional scatter graphs show serum levels vs TFPI2 and CA125 (**A**), and serum TFPI2 levels vs age (**B**), in patients with CCC or EMS in the training and validation sets. EMS, early-stage CCC, and late-stage CCC in the training and validation sets were individually plotted (black dots, EMS in training set; blue dots EMS in validation set; open triangles, early-stage CCC in training set; closed triangles, early-stage CCA in validation set; open circles, late-stage CCC in training set (T); closed circles, late-stage CCC in validation set (V). Dotted lines indicate a predetermined cutoff value (280 pg/mL) for TFPI2 for discrimination of CCC from EMS, and the generally used cutoff value (35 U/mL) for CA125.

Scatter plots of serum levels of TFPI2 and CA125 in patients with CCC and EMS from the training and validation sets are shown in Fig 4A. Spearman’s correlation analysis revealed weak or moderate correlations between TFPI2 level and CA125 level in CCC patients of the training (r_s_ = 0.26) and validation (r_s_ = 0.61) sets. A correlation was also observed in patients with non-CCC EOCs from both sample sets (r_s_ = 0.24 and 0.29). By contrast, no correlation was noted in EMS patients from either sample set (r_s_ = -0.07 and 0.08).

Applying cutoff values of 35 U/mL for CA125 and 280 pg/mL for TFPI2, most patients with late-stage CCC [91% (10/11) and 100% (3/3) in the training and validation sets, respectively] were positive for both CA125 and TFPI2. Of patients with early-stage CCC, 50.0% (9/18 and 2/4) were positive for both biomarkers, and 22.2% (4/18) and 25.0% (1/4) were negative for TFPI2 but positive for CA125, in the training and validation sets, respectively. By contrast, 5 of 6 early CCC patients with CA125 below 35 U/mL were positive for TFPI2. These results indicated a complementary association between CA125 and TFPI2 that increased the sensitivity for detection of patients with early-stage CCC. The sensitivity of the combined assay with CA125 and TFPI2 for detection of early-stage CCC was 96.6% (17/18) and 75.0% (3/4) in the training and validation sets, respectively.

### Effect of age and menstruation periods on serum TFPI2 level

TFPI2 is abundantly expressed in placenta, and its levels are significantly elevated in the serum of pregnant women [13]. Therefore, we investigated the effect of age or menstruation cycle on serum TFPI2 level. As shown in Fig 4B, there were no trends common between the training and validation sets for effect of age on serum TFPI2 level when each group (benign ovarian tumors, CCC and non-CCC EOCs) was considered. Also, in a subset of samples of the validation set (20 patients with ovarian diseases and 13 patients with Ut diseases), we compared serum levels of TFPI2 with days from the last menstrual period at the time of sample collection. Unclear association was noted between serum TFPI2 levels and menstrual periods in these patients (S4 Fig).

## Discussion

The current study demonstrates that measurement of serum TFPI2 level is a promising tool for preoperative testing to predict patients with CCC, a malignant subtype of EOC. We provide clinical evidence that serum TFPI2 can discriminate patients with CCC from patients with ovarian benign tumors, as well as other EOC subtypes. We examined the predictive potential of serum TFPI2 for CCC in a training set, and confirmed its reproducibility in an independent validation set.

CCC is a highly lethal histological subtype of EOC. Although the poor prognosis of ovarian CCC is thought to result primarily from its highly chemoresistant nature [4], some studies have reported aggressive behavior even at an early stage [19, 20].

To date, the early detection of ovarian CCC has been hindered by the absence of effective serum biomarkers. Previous studies have failed to identify a biomarker panel with significantly improved performance over the traditional EOC biomarker, CA125 [8]. However, CA125 exhibits low sensitivity for early disease [21, 22], which represents the majority of CCC [17, 18]. Furthermore, CA125 also suffers from low specificity: elevated levels are seen in many benign abdominal diseases including EMS [22]. Thus, CA125 is not efficient for diagnosis of CCC.

Serum TFPI2 is a novel biomarker for ovarian CCC. This biomarker is highly specific for ovarian CCC, because unlike CA125, its levels were barely elevated in patients with EMS or other EOC subtypes. The CCC-specific elevation of serum TFPI2 was consistent with the mRNA expression pattern in tumor tissues (Fig 1 and S1 Fig). This finding suggests that the up-regulation of *TFPI2* gene in tumor tissue is responsible for the serum level of TFPI2. By contrast, in other cancers, expression of *TFPI2* is reduced or absent following aberrant methylation of its promoter region [23–27]. In addition, we recently obtained preliminary data suggesting that serum TFPI2 levels are not elevated in patients with clear cell–type renal cell carcinoma (data not shown). Therefore, we speculate that with the exception of ovarian CCC, diseases that increase serum TFPI2 level are very rare; however, very few reports have assessed the serum level of TFPI2 is investigated in other kinds of cancer patients. Our data showed that serum TFPI2 is a good biomarker for discriminating ovarian CCC from other ovarian diseases, but further investigation is needed to determine whether TFPI2 can distinguish between ovarian CCC and cancers in other tissues.

The diagnostic sensitivity of TFPI2 for CCC was comparable to that of CA125 among all EOC patients, but TFPI2 was superior to CA125 in discrimination of CCC patients from other EOC subtypes with high diagnostic specificity and accuracy (Table 3). Therefore, serum TFPI2 is a potential distinguishing indicator for EOC subtypes. Among EOC subtypes, CCC exhibits a weaker response to chemotherapy. Despite advances in platinum-based combination chemotherapy, patients with CCC, especially advanced-stage cancer or recurrent disease, have a worse progression-free survival and overall survival than patients with other EOC subtypes [28]. Preoperative prediction of CCC by serum TFPI2 testing may contribute to selection of personalized surgery or molecularly targeted therapy (such as PI3K/mTOR inhibitor) for this EOC subtype, thereby improving survival of ovarian CCC patients [29].

CCC and endometrioid-type EOC is closely associated with EMS, suggesting that its precursor is EMS [30–32]; consequently, CCC is also known as “endometriosis-associated ovarian cancer” [33]. There is good evidence that EMS is a risk factor for CCC and endometrioid EOCs [34]. CCC accounts for ~70% of all endometriosis-associated ovarian cancers, and is associated with worse prognoses than endometrioid-type cancers [33]. Therefore, it is very important to carefully monitor the transformation from EMS into ovarian CCC. However, CA125 is not suitable for such monitoring, because it has a high false-positive rate in patients with EMS [35]. In fact, there were many EMS patients with serum CA125 levels above 35 U/mL in this study. Diagnostic specificity and accuracy of CA125 in discrimination of CCC patients from EMS patients were very low (below 55%), showing that this biomarker is not effective for monitoring of malignant transformation of EMS. By contrast, the diagnostic specificity and accuracy of TFPI2 were very high (over 90%) (Table 3). Furthermore, we found that most early-stage CCC patients with lower TFPI2 levels (< 280 U/mL) were complementally positive for CA125 (Fig 4A). Thus, we believe that TFPI2, alone or in combination with CA125, would be useful for monitoring the malignant transformation of EMS.

TFPI2, also known as placental protein 5 (PP5) [36, 37], is abundantly produced in placenta and is significantly elevated in the serum of pregnant women [13]. Therefore, we speculated that serum concentration of TFPI2 might vary in association with menstruation or menopause. However, there was no evidence for an effect of age or menstrual period on serum TFPI2 levels (Fig 4B and S4 Fig). Thus, unlike CA125, whose serum concentration varies between phases of the menstrual period [38, 39]. TFPI2 may be robust with respect to menstruation. However, further studies using more many samples are required to confirm these correlations.

Higher TFPI2 levels (> 345 pg/mL) were also observed in a part of patients with serous-type EOC (7/27 and 5/17 in the training and validation sets, respectively) (S1 Table). Of these serous EOC patients, all were at advanced stages (FIGO III or IV), and many had much higher levels of CA125 (> 1,000 U/mL). Therefore, EOC subtype diagnosis should take into account other clinical findings. Overlaps between ovarian CCC and benign ovarian diseases in serum TFPI2 are also barely recognized. However, overlaps are larger in serum CA125 that is currently used as biomarker for ovarian CCC. Ovarian CCC has a poorer prognosis than EOCs of other subtypes. If TFPI2 has the potential to preoperatively detect early stage CCC and those at risk of disease, it would represent an important step in decreasing mortality from CCC.

The major limitation of this study is the small size of validation set. We are now starting a multi-institution clinical study of CCC diagnosis using the improved TFPI2 assay method, the Direct Assay, which is suitable for clinical use because it can rapidly detect serum TFPI2 without sample dilution. Our data however was able to demonstrate that TFPI2 has a high potential of being used as a serum biomarker for simple screening of patients with CCC, facilitating early treatment decisions to improve prognosis of patients with this EOC subtype, and monitoring the malignant transformation of EMS.

## Supporting Information

S1 Fig *TFPI2* mRNA levels in ovarian tumor tissue samples from a subset of patients (n = 93) from the training set. ***TFPI2* mRNA was quantitated by real-time PCR.** Data were normalized against β-actin mRNA levels. Box plots display 25^th^, 50^th^ (median, middle horizontal line), and 75^th^ percentiles; whiskers show min and max values. Abbreviations: BN, benign ovarian diseases; BD, borderline ovarian tumors; Ut tumors, uterine tumors; CCC, clear cell carcinoma; EMS, endometriosis; EMD, endometrioid; UF, uterine fibroids.
(TIFF)

S2 Fig Distribution of CA125 and TFPI2 in serum samples from patients with CCC and other gynecological diseases (prospective validation set, n = 156). CA125 levels in patients with CCC, other ovarian diseases and uterine (Ut) diseases (**A**). Distribution of serum CA125 levels measured before surgery is shown. Serum TFPI2 levels in patients with CCC, other ovarian diseases, and Ut diseases (**B**). Serum TFPI2 levels were measured by the Pre-Diluted Assay. Comparison of CA125 (**C**) and TFPI2 (**D**) levels in patients with EMS, non-EMS, each EOC subtype, various Ut tumors (UF: uterine fibroids, cervical cancer, endometrial cancer), or other diseases. Box plots display 5^th^, 25^th^, 50^th^ (median, middle horizontal line), 75^th^, and 95^th^ percentiles. Statistical analysis was performed by nonparametric Mann-Whitney *U* test.
(TIFF)

S3 Fig Correlation between serum TFPI2 levels measured by the Direct Assay (ordinate) and the Pre-Diluted Assay (abscissa). Plotted values were obtained using both assay methods in a subset (n = 130) of the validation set. The CCC patient with the highest TFPI2 level was excluded.
(TIFF)

S4 Fig Serum levels of TFPI2 vs phase of menstrual period of 20 patients with ovarian diseases and 13 patients with Ut diseases. Cross marks indicated the patients with EMS.
(TIFF)

S1 Table Clinical information of non-CCC patients with higher TFPI2 levels (>345 pg/mL). (XLSX)

S2 Table Comparison of discrimination performances of the Pre-Diluted Assay and the Direct Assay for TFPI2. (XLSX)
