# Supplementary figures and images for: Clinical Significance of Tissue Factor Pathway Inhibitor 2, a Serum Biomarker Candidate for Ovarian Clear Cell Carcinoma
Source: PLoS One. 2016 Oct 31;11(10):e0165609. doi: 10.1371/journal.pone.0165609 (PMC5087914; doi:10.1371/journal.pone.0165609)

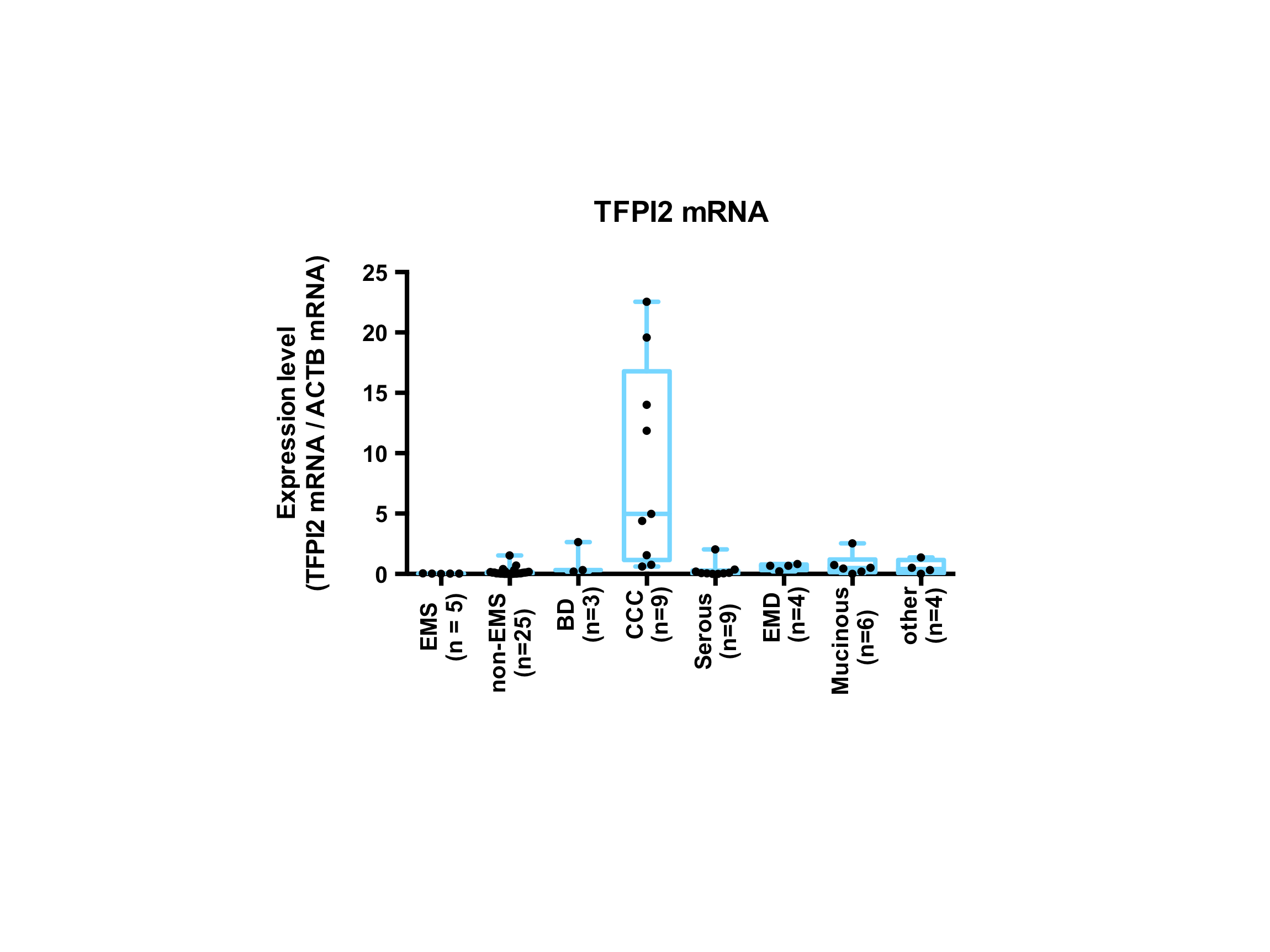

Supplement: S1 Fig — TFPI2 mRNA was quantitated by real-time PCR. Data were normalized against β-actin mRNA levels. Box plots display 25th, 50th (median, middle horizontal line), and 75th percentiles; whiskers show min and max values. Abbreviations: BN, benign ovarian diseases; BD, borderline ovarian tumors; Ut tumors, uterine tumors; CCC, clear cell carcinoma; EMS, endometriosis; EMD, endometrioid; UF, uterine fibroids. (TIFF) [file pone.0165609.s001.tiff]

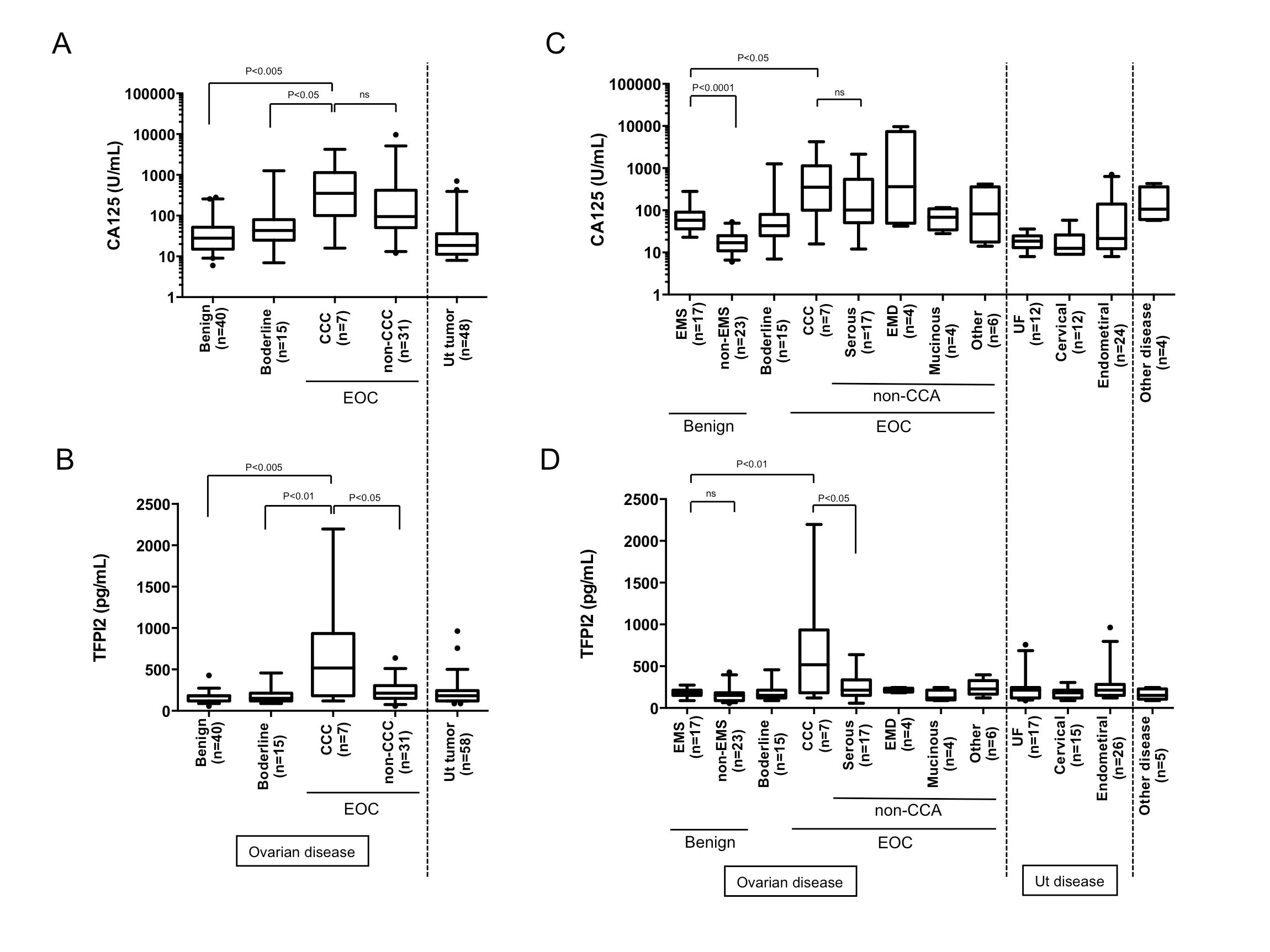

Supplement: S2 Fig — CA125 levels in patients with CCC, other ovarian diseases and uterine (Ut) diseases (A). Distribution of serum CA125 levels measured before surgery is shown. Serum TFPI2 levels in patients with CCC, other ovarian diseases, and Ut diseases (B). Serum TFPI2 levels were measured by the Pre-Diluted Assay. Comparison of CA125 (C) and TFPI2 (D) levels in patients with EMS, non-EMS, each EOC subtype, various Ut tumors (UF: uterine fibroids, cervical cancer, endometrial cancer), or other diseases. Box plots display 5th, 25th, 50th (median, middle horizontal line), 75th, and 95th percentiles. Statistical analysis was performed by nonparametric Mann-Whitney U test. (TIFF) [file pone.0165609.s002.tiff]

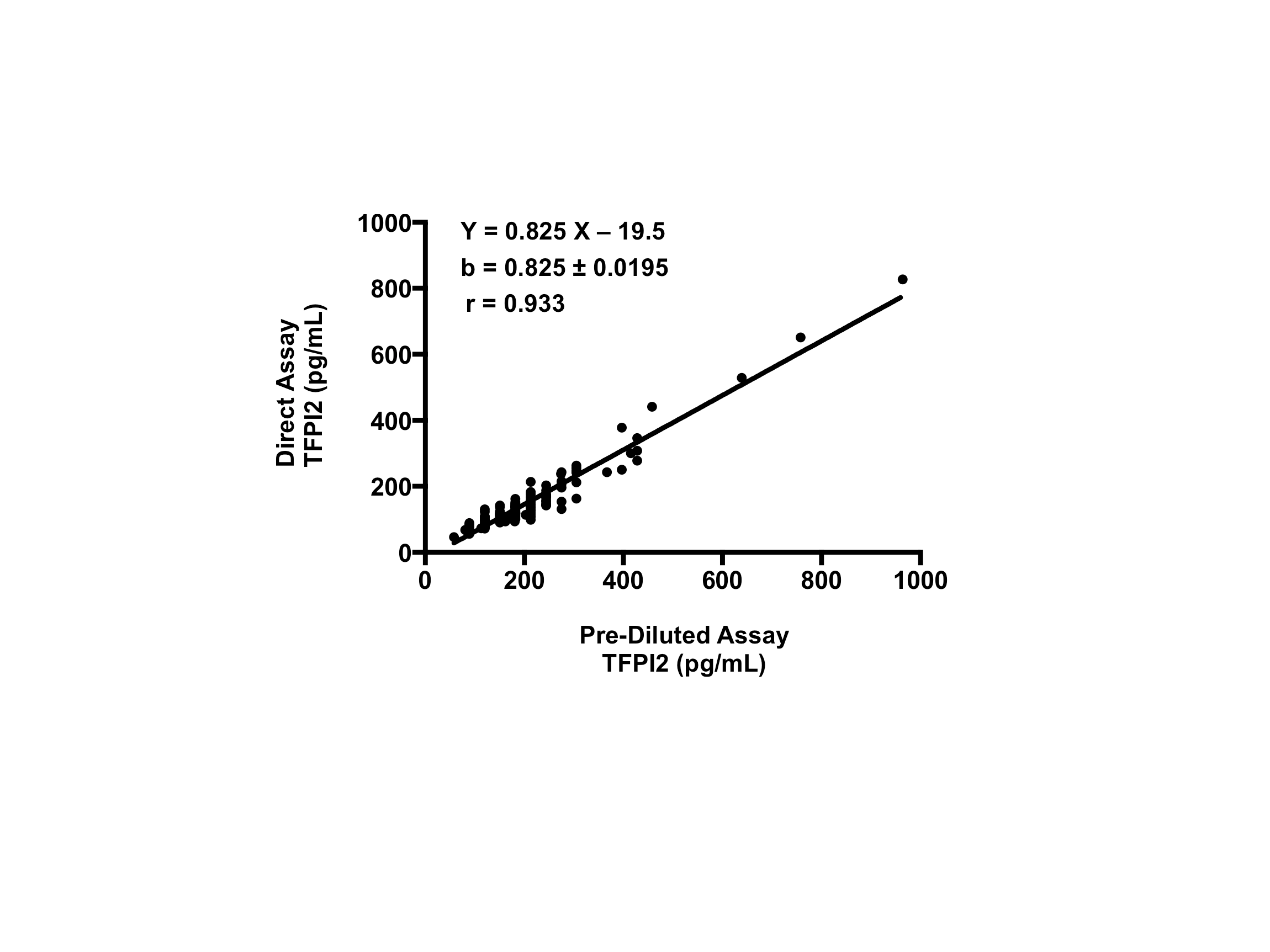

Supplement: S3 Fig — Plotted values were obtained using both assay methods in a subset (n = 130) of the validation set. The CCC patient with the highest TFPI2 level was excluded. (TIFF) [file pone.0165609.s003.tiff]

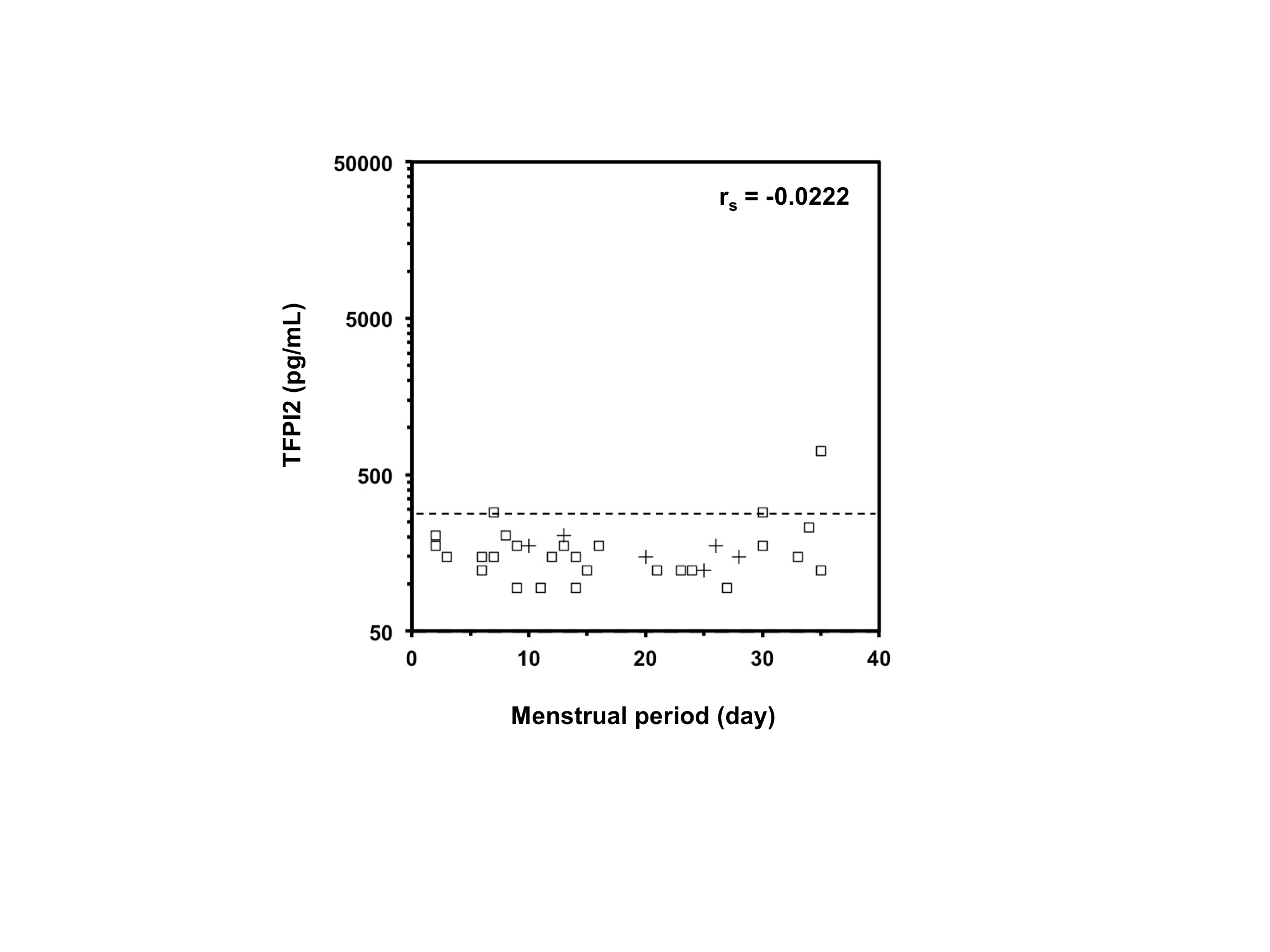

Supplement: S4 Fig — Cross marks indicated the patients with EMS. (TIFF) [file pone.0165609.s004.tiff]
